# Diagnosing Human Fascioliasis Using ELISA Immunoassays at a Tertiary Referral Hospital in Hanoi: A Cross-Sectional Study

**DOI:** 10.3390/tropicalmed7050076

**Published:** 2022-05-17

**Authors:** Huong Nguyen Thu, Veronique Dermauw, Tho Tran Huy, Clémentine Roucher, Pierre Dorny, Hoai Nguyen Thi, Kien Hoang Trung, Thang Dao Van, Binh Do Nhu, Thu Nguyen Kim

**Affiliations:** 1 Department of Microbiology-Parasitology, Faculty of Basic Medicine, Hanoi University of Public Health, Hanoi 11900, Vietnam; nth14@huph.edu.vn; 2 Department of Biomedical Sciences, Institute of Tropical Medicine, 2180 Antwerp, Belgium; vdermauw@itg.be (V.D.); croucher@itg.be (C.R.); pdorny@itg.be (P.D.); 3 National Institute of Malariology, Parasitology and Entomology, Hanoi 12000, Vietnam; huythonimpe@yahoo.com; 4 Department of Virology, Parasitology and Immunology, 9820 Merelbeke, Belgium; 5 Department of Informatic Science, Vietnam Military Medical University, Hanoi 12108, Vietnam; hoaihvqy@gmail.com; 6 Department of Immunology, Vietnam Military Medical University, Hanoi 12108, Vietnam; hoangtrungkienk90@gmail.com; 7 Department of Infectious Disease, Military Hospital 103, Hanoi 12108, Vietnam; thangvmmu@gmail.com; 8 Department of Infectious Disease, Hanoi Medical University, Hanoi 11520, Vietnam

**Keywords:** *Fasciola* spp., human fascioliasis, Vietnam, serologic tests, ELISA

## Abstract

Infections with the zoonotic liver flukes *Fasciola gigantica* and *Fasciola hepatica* may result in severe disease in humans. In Vietnam, an emergence of fascioliasis cases has been observed from the late 1990s onwards. Various diagnostic tools are used in the country, but the agreement between these tools has not been critically evaluated. We aimed to describe the clinical presentation and diagnostic outcomes in fascioliasis patients in Vietnam. A retrospective, cross-sectional study was conducted on the medical records of a group of 145 patients diagnosed with fascioliasis at a tertiary referral hospital in Hanoi. Based on the review, sociodemographic background and clinical presentation were recorded. These patients all received standard routine serologic tests, including internal antibody (Ab)-ELISA, an enzyme-linked immunosorbent assay (ELISA), and commercial coproantigen (cAg)-ELISA. The majority of cases were between 30 and 59 years old (68.3%), and about half of them were male (51.0%). Upper quadrant and epigastric pain were the most commonly reported symptoms (61.4% and 35.2%, respectively). All but one patient had liver lesions upon ultrasound examination (99.3%), and eosinophilia was present in most of the patients (89.7%). A high number of patients were positive in the in-house and the commercial Ab-ELISA (95.9% and 87.4%, respectively), yet only a slight agreement was observed between the two tests (kappa coefficient, 0.06). A further 47.4% of cases were positive for the commercial cAg-ELISA, whereas stool microscopy indicated the presence of *Fasciola* spp. eggs in 25.7% of patients. The current study emphasizes the challenges related to the diagnosis of human fascioliasis in Vietnam.

## 1. Introduction

Fascioliasis is a zoonotic foodborne hepatic disease caused by the digenean trematodes *Fasciola gigantica* and *Fasciola hepatica* [1]. The life cycle of these liver flukes involves freshwater snails of the Lymnaeidae family as intermediate hosts and a wide range of mammals, including cattle, sheep, and also humans as definitive hosts [2]. Transmission of the parasites to humans mainly occurs via ingestion of aquatic vegetables and water, contaminated with metacercariae [3]. After decades of being considered of secondary importance, human fascioliasis, characterized by cholangitis, cholecystitis, liver abscesses, and even death, has now been recognized as an important neglected zoonotic disease with a significant public health burden [4]. Worldwide, an estimated 2.6 million people are infected with *Fasciola* spp., and another 180 million are thought to be at risk for infection [5,6]. 

In Vietnam, the emergence of fascioliasis has taken worrying proportions and has been called a major public health problem by WHO. From 1997 up to 2000, over 500 cases were reported [7], whereas 4585 patients were identified from 2000 to 2006 [8]. Common complaints were upper quadrant pain (87.1%), mild fever (39.8%), upper stomach ache (30.5%) and indigestion (26.1%) [9]. In Vietnam, fascioliasis can be found in all age categories, yet women and children seem more vulnerable to the disease [10]. Here, people typically get infected through the consumption of contaminated submerged water plants such as water convolvulus [10]. 

The diagnosis of fascioliasis is challenging. Classical coprological techniques using microscopy are characterized by a reasonable specificity, yet poor sensitivity, and are unable to detect fascioliasis early [11,12]. Serum antibody (Ab) detection using enzyme-linked immunosorbent assay (ELISA), on the other hand, is considered a specific and sensitive method for fascioliasis diagnosis and allows for earlier detection [11,12]. However, Ab-ELISA cannot differentiate past from current infection [11], and full validation of locally produced kits, e.g., in Vietnam, is currently lacking [13]. In response to these drawbacks, WHO has proposed a combined evaluation of test results to allow differentiation of different stages of the infection, yet interpretation remains challenging [12]. Ultrasound examination might be considered the best method to detect fascioliasis-related lesions. However, ultrasound is not always able to differentiate fascioliasis from other liver diseases, and it is often unavailable in a community setting [12,13]. Another diagnostic tool available, the coproantigen (cAg) detection technique, has a high specificity and sensitivity and allows early detection, as well as differentiation between current and past infection [11]. Unfortunately, it has not been applied in the Vietnamese setting up to now.

Overall, considering that few original data on fascioliasis cases in Vietnam have been published in international journals [14], the aim of the current study was to (i) describe the clinical presentation of a group of patients suspected of suffering from fascioliasis after examination at a tertiary referral hospital in Hanoi, Vietnam, and to (ii) to assess the agreement between the different diagnostic tools available.

## 2. Materials and Methods

### 2.1. Study Area and Design

Patients visiting peripheral clinics in North Vietnam, who were suspected of suffering from fascioliasis based on clinical presentation, were advised to receive a more thorough examination at the clinical department of the tertiary referral center in Hanoi.

From June 2011 until December 2013, data were gathered on all patients receiving the confirmatory diagnosis of fascioliasis at the tertiary referral hospital in Hanoi. Sociodemographic characteristics of the patients were collected, as well as information on clinical presentation. Results of the following standard tests used at the hospital for fascioliasis diagnosis were gathered: (i) ultrasound imaging of the abdomen, (ii) stool microscopy for *Fasciola* spp. eggs, and serum analysis for Ab against *Fasciola* spp. using an in-house ELISA, (iii) serum liver enzyme levels, and (iv) differential white blood cell count. Furthermore, in the context of the current study, the following additional tests were performed on the samples obtained earlier for the routine tests: (i) serum analysis for Ab against *Fasciola* spp. using a commercial ELISA, and (ii) fecal examination for *Fasciola* spp. Ag using a commercial copro-ELISA.

### 2.2. Sample and Data Collection

Patients were selected for the study on archival records with sufficient information on clinical and subclinical diagnoses. As part of standard testing, an in-house Ab-ELISA test was run on serum samples and microscopy on stool samples (see further below), the results of which were collected. The sample bank checked that sufficient serum for each patient and stool samples stored at −20 °C were available for further analysis. Furthermore, the investigator collected sociodemographic data (e.g., age, gender, employment) and recorded symptoms through archived medical records. All of these patients underwent a two-dimensional abdominal ultrasound investigation (SDU 350XL, Shimadzu, Kyoto, Japan). The conclusion as noted by the sonographer was collected for this study. A positive ultrasound investigation was defined as the presence of one or more nonhomogenous hypodense areas in the liver, which may include small, clustered lesions in liver segments V and VI with slight contrast, with normal enhanced liver tissue within the lesion.

### 2.3. Sample Analysis

#### 2.3.1. Measurement of Serum IgG Ab against *Fasciola* spp.

Serum samples were analyzed for IgG Ab against *Fasciola* spp. using an in-house Ab-ELISA, as part of the standard examination, while within the context of the current study, the samples were analyzed using a commercial Ab-ELISA as well. The in-house Ab-ELISA used partially purified excretory/secretory (E/S) antigens produced from adult *F. gigantica* recovered from bovine livers [15]. Worms were washed in sterile saline and in an enriched Roswell Park Memorial Institute 1640 (RPMI-1640) medium to remove host antigens. Next, 50 worms were incubated for 12 h at 37 °C under 5% CO_2_ in a 150 mL RPMI 1640 medium containing penicillin and streptomycin. The medium was centrifuged at 10,000× *g* for 20 min at 4 °C before being semi-purified by running on a minipore column (Shanghai Minipore Industrial, Shanghai, China). A fraction of more than 5 kDa was resuspended in phosphate-buffered saline (PBS) with a protease inhibitor cocktail (Sigma-Aldrich, St. Louis, MO, USA) at a final concentration of 1 mg/mL till used. Protein concentration was determined by the method of Bradford [16]. The antigen (Ag) was diluted at 25 μg/mL in carbonate buffer before being coated on Nunc Maxisorp microtiter plates. Target concentrations of Ag and Ab in ELISA were established by checkerboard titration.

Serum from patients and controls was applied at a 1:200 concentration. An anti-human IgG-peroxidase goat Ab (Sigma-Aldrich) was used as the detecting Ab at a 1:6000 dilution. O-phenylenediamine (OPD) (Sigma-Aldrich) was used as the substrate. Incubations were performed at 37 °C for one hour. Then, the wells were washed 3 times with washing solution (PBS 1X). The plates were read after stopping the reaction with H_2_SO_4_ using a spectrophotometer at 492 nm, with a reference at 630 nm. On each plate, eight negative controls were used for cut-off determination (mean + three standard deviations) and two positive controls for quality control. Negative controls are serum samples obtained from perfectly healthy individuals free from any parasites. The positive controls were samples collected from human fascioliasis patients with the clinically confirmed infection that was stored for this study.

The commercial Ab-ELISA using MM3 as *Fasciola hepatica*-antigen coating was performed according to the manufacturer’s instructions (Bio-X Diagnostics, Rochefort, Belgium). The test was delivered with human conjugate and was internally validated by the manufacturer, using human samples. The serum samples were diluted once to 1:100 using the diluent buffer in the kit. Avoid using hemolyzed samples or those containing coagulum.

#### 2.3.2. Blood Count and Biochemical Testing

Furthermore, as part of the routine testing, a differential white blood cell count was performed to determine the percentage of eosinophils (Mek-2222, Nihon Kohden, Tokyo, Japan). Eosinophilia was defined as an increase in eosinophilic leukocytes to more than 6% of white blood cells counted [17]. In addition, samples were analyzed for levels of serum aspartate aminotransferase (AST) and serum alanine aminotransferase (ALT) (AU680, Beckman Coulter, Brea, CA, USA). Elevated AST and ALT levels were defined as AST and ALT levels above 35 IU/L and 56 IU/L, respectively [17].

#### 2.3.3. Stool Examination

For standard testing, three fresh stool samples collected at three different times were microscopically examined for eggs of *Fasciola* spp. using the formalin-ether sedimentation technique [18]. Finally, within the context of the current study, stool samples were also analyzed for excretory/secretory Ag using a commercial MM3-copro-Ag-ELISA (cAg-ELISA) [19]. This test was developed using Ab against *F. hepatica* and was performed according to the manufacturer’s instructions, using a cut-off of 0.3 (Bio-X Diagnostics, Rochefort, Belgium). This test was developed for animal samples; however, as essentially the same parasite Ag is being detected in animal and human samples, no-species specific optimization is necessary. Stool samples were diluted in the provided dilution buffer before the analysis. In this study, we excluded all the samples that showed coinfection with multiple parasites.

### 2.4. Statistical Analysis

The data were entered in Excel (Microsoft Office Excel, 2010). There were missing values (mv) for the following variables: occupation (*n* = 1), stool microscopy (*n* = 1), commercial Ab-ELISA (*n* = 2), and copro-Ag-ELISA (*n* = 10). A descriptive statistical analysis was performed for all variables, while the agreement between diagnostic test results was calculated in the subset of suspected fascioliasis cases with test results available for all diagnostic tools. The level of agreement was expressed by means of the Cohen kappa statistic [20], using the following classification: “slight” (kappa value: 0.01–0.20), “fair” (0.21–0.40), “moderate” (0.41–0.60), “substantial” (0.61–0.80), or “almost perfect” agreement (>0.81) [21]. Calculation of the kappa statistic and its 95% confidence intervals (95% CIs) was conducted using the KAPPA test function (package “psych”). All statistical procedures were conducted using R, version 3.5.1 [22].

### 2.5. Ethics Statement

All people described in this research signed written informed consent for the publication of the case details, and the protocol was approved by the Ethical Review Committee of the National Institute of Malariology, Parasitology, and Entomology (No. 228/QD-VSR; date: 16 May 2011). This study was also conducted using good clinical practice following the Declaration of Helsinki and its later amendments or comparable ethical standards.

## 3. Results

The mean age of the 145 suspected fascioliasis patients was 42.4 ± 14.1 years, with more than two-thirds of the studied group being between 30 and 59 years old (68.3%, 99/145) (Table 1). Slightly above half of the patients were male (74/145, 51.0%), and the majority were farmers (63/144, 43.8%, 1 mv) or service employees (34/144, 23.6%, 1 mv). Most patients originated from the North Central (77/145, 53.1%) or the Red River Delta regions (50/145, 34.5%).

The most commonly reported symptoms were upper quadrant pain (89/145, 61.4%), followed by epigastric pain (51/145, 35.2%) (Table 2). Nine patients reported both symptoms (9/145, 6.2%). Other signs and symptoms were fever (16/145, 11.0%), extreme fatigue (6/145, 4.14%), and digestive disorders (1/145, 0.69%). Nine patients reported both fever and epigastric or upper quadrant pain (9/145, 6.2%). For all but one patient, liver lesions were observed upon ultrasound examination (144/145, 99.3%). In a vast majority, eosinophilia was present (130/145, 89.7%), with the mean percentage of circulating eosinophils being 32.3 ± 19.1, and 40.0% (58/145) of patients presenting with very high eosinophil percentages (40% and above). High AST and ALT levels were observed in 73.8% (107/145) and 36.6% (53/145) of patients, respectively, with mean AST and ALT levels of 57.1 ± 61.2 and 61.9 ± 64.7 IU/L, respectively.

Serum samples of nearly all patients were positive in the in-house Ab-ELISA (95.9%, 139/145), while 87.4% were positive in the commercial Ab-ELISA (125/143, 2 mv). Stool samples of 47.4% of patients were positive in the cAg-ELISA (64/135, 10 mv), whereas in 25.7% of patients (37/144, 1 mv), *Fasciola* spp. eggs were observed upon microscopic examination. Detailed test results for the subset of suspected fascioliasis cases with all the diagnostic tools run (*n* = 132, 13 mv) are shown in Table 3.

For those patients, only a slight agreement was observed between the in-house and the commercial Ab-ELISA (kappa value, 0.06; 95%CI, 0–0.48), mainly due to patients having a positive result in the in-house Ab-ELISA but a negative result in the commercial Ab-ELISA (82.4%, 14/17 patients in disagreement). The agreement between the cAg-ELISA and stool microscopy was moderate, with a kappa value of 0.44 (95%CI, 0.29–0.60). The in-house Ab-ELISA was able to correctly identify patients positive on stool microscopy in 97.1% of cases (34/35), whereas the commercial Ab-ELISA could identify all cases positive on microscopy (35/35). The cAg-ELISA correctly detected 88.6% of cases positive on stool microscopy (31/35).

## 4. Discussion

Up to now, few original data on fascioliasis cases in Vietnam have been published in international journals [7,23]. Some authors [10,13,14] have reviewed local reports, indicating a quick rise in human fascioliasis cases from the late 1990s onwards, the reason for which remains unclear up to now. Several hypotheses have been put forward, such as increased availability of more sensitive diagnostic methods, higher awareness among health professionals in the country, and altered climatic conditions and behavioral aspects [10,14]. Further studies are essential to monitor the disease and fully unravel the mechanism behind any changes in disease incidence.

In this study, 145 patients were clinically diagnosed with fascioliasis as described, all of whom were examined at the study hospital facilities from 2011 up to 2013 onwards. In contrast to an earlier study [7] examining 500 patients from 1997 up to 2000 with a majority (66.6%) of patients being female, an equal number of female and male patients was observed in our study. A higher prevalence of seropositivity in females (6.4 vs. 5.2%) was also observed by other authors [23] in patients tested for fascioliasis at Ho Chi Minh City and in local reports [10,13,14]. In the same original study and reviews [7,14,23], the majority of patients were between 20–30 and 50–60 years of age, which is in line with the findings of the current study.

Almost half of the examined patients were farmers, and the vast majority originated from the North Central or the Red River Delta regions, the latter in agreement with earlier findings indicating a high number of cases in the central and, to a lesser degree, northern provinces [10,13]. However, the real geographical distribution of cases might be profoundly different from that reported here and in earlier studies, as patients in remote rural areas might never reach the referral hospitals.

In agreement with earlier work, the vast majority of patients suffered from upper quadrant pain, while fewer patients had fever [7,9]. In contrast, fever (49.0–52.9%) and fatigue (68.4%–79.4%) seemed to be much more prevalent in fascioliasis cases in a community setting in Central Vietnam [24]. In the current study, eosinophilia was present in a high percentage of patients, in line with the hospital study in Ho Chi Minh City [7], and higher than in infected people in the community study (52.9–57.4%) [24]. Levels of AST and ALT were elevated in a high percentage of patients, with mean levels being higher than those reported in a study in Egypt comparing fascioliasis cases with healthy controls [25].

All patients in the studied group were examined by medical doctors at the referral hospital and declared to suffer from fascioliasis, mainly based on positive ultrasound and/or positive serology (in-house Ab-ELISA). According to the combined evaluation of serology and coprology results, as proposed by WHO [12], three patients would have been categorized as having either “no infection” or “resolved infection” judging from the negative results of coprology and in-house Ab-ELISA tests. In contrast, 17 patients would have been categorized the same way, based on the commercial Ab-ELISA results. A further two and one patients, based on the positive in-house or commercial Ab-ELISA results, respectively, and negative coprology, would have been classified as having “acute or ectopic infection”, “resolved infection”, “biliary obstruction”, or presenting with “intermittence of egg shedding”; a group of patients for whom indeed further categorization is difficult. Another 35 and 36 patients (for the in-house or commercial Ab-ELISA, respectively) would have been regarded to suffer from “liver infection”, because of positive coprology combined with positive serology, while 104 and 88 patients, respectively, would have been classified as having “chronic liver infection” due to positive coprology combined with negative serology.

Overall, the proposed approach by the WHO would indicate that for at least some patients in the current study, the diagnosis of fascioliasis was false. However, it is unclear whether this classification is valid using the commercial and in-house Ab-ELISA applied in the current study. In the current study, we did not differentiate species; however, as mentioned above, human fascioliasis in Vietnam can be caused by *F. gigantica*, as well as *F. gigantica/F. hepatica* hybrids. Unfortunately, current commercial Ab-ELISAs are generated from *F. hepatica* antigens, and it is thus not clear whether this test has the same performance for antibody detection against *F. gigantica* or hybrids, although results from experimental infections in animals are supporting the use of MM3 for diagnosis of both species in serum (Ab-ELISA) and stool (cAg-ELISA) [26]. The in-house Ab-ELISA, on the other hand, was developed specifically with *F. gigantica* Ag, which could explain the disparate results with the commercial test, yet further evaluation of test performance is needed.

## 5. Conclusions

A total of 145 suspected fascioliasis cases diagnosed in a tertiary referral hospital in Hanoi, were described, most of which suffered from upper quadrant pain. The results of the present study indicated that there was poor agreement between the in-house and the commercial Ab-ELISA tests, whereas the cAg-ELISA seemed to detect more cases than traditional microscopy. All techniques, however, require further validation for diagnosis of human fascioliasis caused by *F. gigantica* and *F. gigantica/F. hepatica* hybrids. Suspected cases should be examined according to the national guidelines for diagnosis of fascioliasis. These guidelines should be updated once further information is available on the test performance of the tests used in the current study.

## Figures and Tables

**Table 1:** Sociodemographic characteristics of suspected fascioliasis patients examined at a tertiary referral hospital, Hanoi, Vietnam (2011–2013) (*n* = 145).

Parameter	Count	Percentage (%)
Age		
7–15	4	2.8
16–29	25	17.2
30–39	34	23.5
40–49	35	24.1
50–59	30	20.7
above 60	17	11.7
Gender, male	74	51.0
*Occupation* (1 mv)		
Farmer	63	43.8
Services employee	34	23.6
Unemployed	22	15.3
Student	8	5.6
Teacher	6	4.2
Driver	5	3.5
Fisherman	4	2.8
Soldier	2	1.4
*Region*		
North, Red River Delta *	50	34.5
North, North East ^†^	16	11.0
North, North West ^‡^	1	0.69
Central, North Central ^§^	77	53.1
Central, South Central Coast ^‖^	1	0.69

mv = missing value; * Hanoi (*n* = 22), Bac Ninh (*n* = 7), Vinh Phuc (*n* = 7), Hung Yen (*n* = 4), Ha Nam (*n* = 4), Nam Dinh (*n* = 2), Hai Phong (*n* = 2), Ninh Binh (*n* = 1), Thai Binh (*n* = 1); ^†^ Bac Giang (*n* = 11), Guang Ninh (*n* = 3), Lang Son (*n* = 1), Thai Nguyen (*n* = 1); ^‡^ Yen Bai (*n* = 1); ^§^ Nghe An (*n* = 39), Thanh Hao (*n* = 22), Ha Tinh (*n* = 14), Quang Binh (*n* = 1), Quang Tri (*n* = 1); ^‖^ Quang Nam (*n* = 1).

**Table 2:** Clinical features in suspected fascioliasis patients examined at a tertiary referral hospital, Hanoi, Vietnam (2011–2013) (*n* = 145).

Parameter	Number Positive	Percentage (%)
*Signs and symptoms*		
Upper quadrant pain	89	61.4
Epigastric pain	51	35.2
Fever	16	11.0
Extreme fatigue	6	4.1
Digestive disorders	1	0.69
*Blood analysis*		
Eosinophilia (>6%)	130	89.7
Elevated AST (>35 UI/L)	107	73.8
Elevated ALT (>56 UI/L)	53	36.6
*Ultrasound*		
Presence of liver lesions	144	99.3

**Table 3:** Diagnostic tool results in suspected fascioliasis patients examined at a tertiary referral hospital, Hanoi, Vietnam, with complete test results for all available diagnostic tools (2011–2013) (*n* = 132, 13 missing values).

Diagnostic Test					
Serum		Stool			
Ab-ELISA (in-House)	Ab-ELISA (Commercial)	cAg-ELISA	Microscopy	*n*	%
+	+	+	+	30	22.7
+	+	+	-	29	22.0
+	+	-	+	4	3.0
+	+	-	-	51	38.6
+	-	+	+	0	0.0
+	-	+	-	3	2.3
+	-	-	+	0	0.0
+	-	-	-	11	8.3
-	+	+	+	1	0.8
-	+	+	-	0	0.0
-	+	-	+	0	0.0
-	+	-	-	2	1.5
-	-	+	+	0	0.0
-	-	+	-	0	0.0
-	-	-	+	0	0.0
-	-	-	-	1	0.8
Total test positive					
128/132 (97.0%)	117/132 (88.6%)	63/132 (47.7%)	35/132 (26.5%)		

## Data Availability

The data that support the findings of this study are available from the corresponding author, upon reasonable request. If you have concerns about sharing the data, please contact nhubinh.do@vmmu.edu.vn.
